# SHAP and LIME: An Evaluation of Discriminative Power in Credit Risk

**DOI:** 10.3389/frai.2021.752558

**Published:** 2021-09-17

**Authors:** Alex Gramegna, Paolo Giudici

**Affiliations:** Department of Economics and Management, University of Pavia, Pavia, Italy

**Keywords:** SHAP (shapley additive exPlanations), credit risk, default, clustering, explainable artificial intelligence (XAI)

## Abstract

In credit risk estimation, the most important element is obtaining a probability of default as close as possible to the effective risk. This effort quickly prompted new, powerful algorithms that reach a far higher accuracy, but at the cost of losing intelligibility, such as Gradient Boosting or ensemble methods. These models are usually referred to as “black-boxes”, implying that you know the inputs and the output, but there is little way to understand what is going on under the hood. As a response to that, we have seen several different Explainable AI models flourish in recent years, with the aim of letting the user see why the black-box gave a certain output. In this context, we evaluate two very popular eXplainable AI (XAI) models in their ability to discriminate observations into groups, through the application of both unsupervised and predictive modeling to the weights these XAI models assign to features locally. The evaluation is carried out on real Small and Medium Enterprises data, obtained from official italian repositories, and may form the basis for the employment of such XAI models for post-processing features extraction.

## 1 Introduction

Probability of default (PD) estimation is an issue which banks and other financial institutions have been confronting with since the dawn of credit. Systems and methodologies evolved as knowledge and technology did, but it wasn’t until recently that the incredible steps forward made in IT gave a real shake to the way it was performed by the industry. At first, incumbents institutions resisted the application of new paradigms, which favored the emergence of a growing number of Fintech startups whose purpose is to provide an estimation of the creditworthiness of people and firms alike, and make it so that this estimation is the most high fidelity as possible.

To be able to give such estimation, these firms of course leverage new and diverse sources of data, take advantage of innovations in regulatory framework concerning financial data (e.g. European PSD2 (European Commission, (2015)) and exploit the far higher predictive power that some of the newly implemented algorithms offer with respect to traditional methods. The increase in prediction power of new algorithms, though, takes a toll on explainability, since the models are now so complex that it is close to impossible to establish clear links between the inner workings of the model and the given output. This surely represents a problem and hinders their diffusion, other than raising a series of ethical and regulamentary issues, which are starting to be addressed (see, for example European Commission (2020)).

To solve this trade-off, the concept of eXplainable AI (XAI) emerged introducing a suite of machine learning (ML) techniques that produce models that offer an acceptable trade-off between explainability as well as predictive utility and enables humans to understand, trust and manage the emerging generations of AI models. Among the emerging techniques, two frameworks have been widely recognized as the state-of-the-art in eXplainable AI and those are:

• the Lime framework, introduced by (Ribeiro et al., 2016)
• SHAP values, introduced by (Lundberg and Lee, 2017).

In finance, interpretability is especially important because the reliance of the model on the correct features must be guaranteed; yet, there aren’t many studies focusing on the application of XAI in this specific context (Bussmann, 2020). propose a XAI model based on Shapley values applied in the context of loan decisions regarding SME seeking for financing through P2P platforms, whereas the research by (Ariza-Garzón et al., 2020) aim to assess the predictive capacity of several ML models in the context of P2P lending platforms’ credit scoring, after that applying the Shapley method to provide explainability to the prediction. The most interesting precedent is perhaps the research of (HadjiMisheva et al., 2021), where the authors explore the utility of both SHAP and Lime frameworks in the context of credit risk management, outlining the practical hurdles in applying these techniques to several different kinds of ML algorithms as well as proposing solutions to the challenges faced.

Our study aims to compare SHAP and LIME frameworks by evaluating their ability to define distinct groups of observations, employing the weights assigned to features through their local interpretability algorithm as input space for unsupervised approached and a supervised one. We do this building our approach on one of the best performing, yet complex, supervised learning algorithm, XGBoost (Chen and Guestrin, 2016), employed to predict the probability of default of italian Small and Medium Enterprises.

## 2 Methodology

### 2.1 LIME

Locally Interpretable Model Agnostic Explanations is a post-hoc model-agnostic explanation technique which aims to approximate any black box machine learning model with a local, interpretable model to explain each individual prediction (Ribeiro et al., 2016). The authors suggest the model can be used for explaining any classifier, irrespective of the algorithm used for predictions as LIME is independent from the original classifier. Ultimately, LIME works locally which means that it’s observation specific and, just like SHAP, it will provide explanations for the prediction relative to each observation. What LIME does is trying to fit a local model using sample data points that are similar to the observation being explained. The local model can be from the class of interpretable models such as linear models, decision trees, etc. The explanations provided by LIME for each observation x is obtained as follows:

$$\Phi \left(x\right)=argmin_{g\in G}L\left(f,g,\pi_{x}\right)+\Omega \left(g\right)$$ (1)

where *G* is the class of potentially interpretable models such as linear models and decision trees,

*g* ∈ *G*: An explanation considered as a model.

$f:R^{d}\to R$.

*π*_*x*_(*z*): Proximity measure of an instance *z* from *x*.

*Ω*(*g*): A measure of complexity of the explanation *g* ∈ *G*.

The goal is to minimize the locality aware loss *L* without making any assumptions about *f*, since a key property of LIME is that it is model agnostic. *L* is the measure of how unfaithful *g* is in approximating *f* in the locality defined by *π*(*x*).

### 2.2 SHAP

The SHAP framework, proposed by (Lundberg and Lee, 2017) adapting a concept coming from game theory (Lloyd, 1952), has many attractive properties. In this framework, the variability of the predictions is divided among the available covariates; this way, the contribution of each explanatory variable to each point prediction can be assessed regardless of the underlying model (Joseph, 2019).

From a computational perspective, SHAP (short for SHapley Additive exPlanation) returns Shapley values expressing model predictions as linear combinations of binary variables that describe whether each covariate is present in the model or not. More formally, the SHAP algorithm approximates each prediction *f*(*x*) with *g* (*x*′), a linear function of the binary variables *z*′ ∈{0,1}^*M*^ and of the quantities $\phi_{i}\in R$, defined as follows:

$$g\left(z^{\prime }\right)=\phi_{0}+\overset{M}{\underset{i=1}{\sum }}\phi_{i}z_{i}^{\prime },$$ (2)

where *M* is the number of explanatory variables.

(Scott et al., 2018) has shown that the only additive method that satisfies the properties of *local accuracy*, *missingness* and *consistency* is obtained attributing to each variable $x_{i}^{\prime }$ an effect *ϕ*_*i*_ (the Shapley value), defined by:

$$\phi_{i}\left(f,x\right)=\underset{z^{\prime }\subseteq x^{\prime }}{\sum }\frac{|z^{\prime }|!\left(M-|z^{\prime }|-1\right)!}{M!}\left[f_{x}\left(z^{\prime }\right)-f_{x}\left(z^{\prime }\backslash i\right)\right]$$ (3)

where *f* is the model, *x* are the available variables, and *x*′ are the selected variables. The quantity *f*_*x*_ (*z*′) − *f*_*x*_ (*z*′ \ *i*) expresses, for each single prediction, the deviation of Shapley values from their mean: the contribution of the *i*-th variable.

Intuitively, Shapley values are an explanatory model that locally approximate the original model, for a given variable value *x* (*local accuracy*); with the property that, whenever a variable is equal to zero, so is the Shapley value (*missingness*); and that if in a different model the contribution of a variable is higher, so will be the corresponding Shapley value (*consistency*).

### 2.3 Evaluation Approaches

While LIME and SHAP have similar behaviour in that they both obtain parameters for feature contribution at the observation level (local explanation), they do differ in the algorithm which leads to such outcome. In order to see which approach is better in detecting variables’ contribution at the local level, we attempt an unsupervised approach and verify if it is possible to cluster observation employing a dissimilarity matrix built on LIME weights and SHAP values, employing standardized Euclidean distance as the basis for clustering.

More formally, we define the pairwise distance *d*_*i*,*j*_ as:

$$d_{i,j}=\left(x_{i}-x_{j}\right)\Delta^{-1}{\left(x_{i}-x_{j}\right)}^{\prime }$$ (4)

where **Δ** is a diagonal matrix whose *i*-th diagonal element contains the standard deviation. The distances can be represented by a *N* × *N* dissimilarity matrix **D** such that the closer two observations *i*, *j* are in the Euclidean space, the lower the entry *d*_*i*,*j*_.

On the similarity matrix we perform a classical K-means clustering (as defined by (MacQueen, 1967)) and, to represent the connectivity approach and not confine ourselves to the convex clusters originated by K-means clustering, we also run a spectral clustering algorithm, as outlined in (Ng et al., 2001). This is done for both dissimilarity matrices computed on LIME weights and SHAP values. We then look for the best number of clusters *K* using measures that assess clusters’ internal cohesion and external separation, namely the Silhouette (Rousseeuw, 1987) and the Davies–Bouldin index (Davies and Bouldin, 1979). Other than using unsupervised tool to devise groups out of XAI models parameters, we run as well a supervised learning algorithm (Random Forest, as in (Breiman, 2001)) on XAI parameters to see how they perform as input in predicting default, which was the problem we started the analysis with. We compare the two predictive models, one for Lime weights and one for SHAP values, through AUROC (Bradley, 1997). This way, we have a thorough perspective on the discriminative power of eXplainable AI-assigned feature weights.

## 3 Application

### 3.1 Data

Data on italian SME is obtained through the Bureau van Dijk database, which sources data directly from Italian chamber of commerce. We employed some techniques to deal with the strongly unbalanced classes (e.g. *Lin et al. (2017)* approach) (Lin et al., 2017) and to remove time-specific factors. More specifically, we worked on data encompassing the last 6 years, comprising more than 2 millions SME observations, we kept all the defaulted cases and, for the not-defaulted ones, we randomly sampled a group of observation to maintain as they were (about 10,000 for each year), while with the remaining we built 5,000 clusters per year and employed the medoids as input observations. This brought down class imbalance from about 100 : 1 to 5 : 1, allowing the model to better frame risk patterns and give more amplitude to probability estimation.

The above procedure led us to a dataset with about 139,000 observations, with 27,200 defaults. We split the dataset assigning 70% of observation to the trainig set and 30% to the test set using stratified partitioning, run the chosen supervised algorithm (*XGBoost*), then apply LIME and SHAP to the test set to get the respective parameters; these are extracted for both methods as linear combinations of variables contributions’, therefore are similar in magnitude and behaviour and thus comparable through our methodology.

## 3.2 Results

**FIGURE 1:**
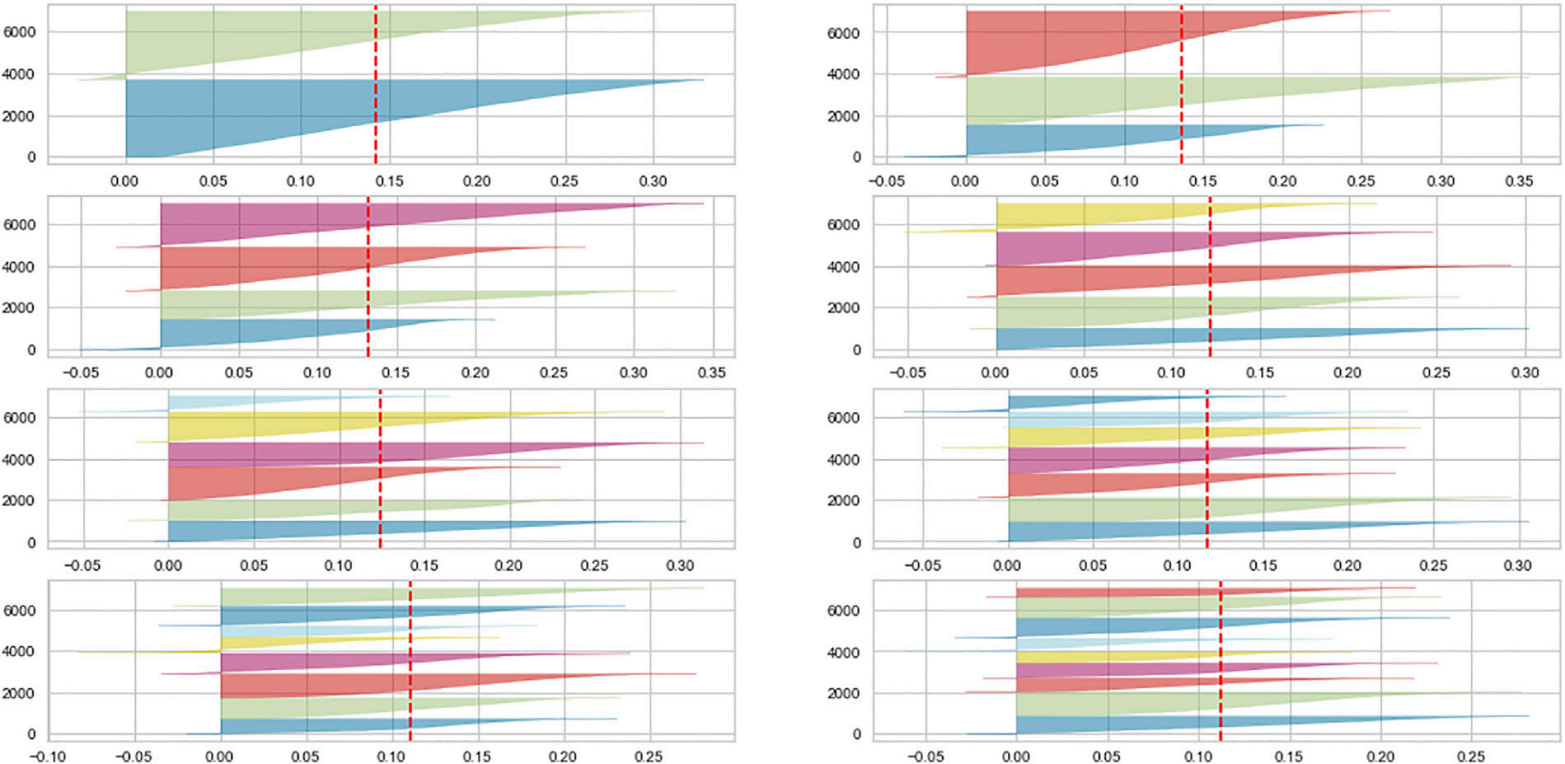
Silhouette plot for LIME data clustering

To select the number of clusters K we examine the silhouette plot (Rousseeuw, 1987) of both generated dataset, for K from 2 to 9. Either for SHAP or LIME, the number of clusters which maximizes the silhouette score is two, coherently with the problem at hand (default prediction); we can see this by looking at the silhouette scores represented by the vertical red dashed line, which is higher for the plot with two clusters, and also from the part of the clusters who enter the *X* axis negative score, which increase as we increase the number of clusters. We show in Figure 1 the silhouette graph for LIME data clustering , being the one for SHAP being basically identical, albeit with a higher average silhouette score, as we are addressing in the following lines.

We therefore perform K-means clustering and Spectral clustering on the two sets of data, with the aim of evaluating the goodness of fit of the clustering approach on XAI parameters through Silhouette score and Davies–Bouldin index (DBI). Here, the higher the Silhouette score, the better externally separated and internally cohese are the clusters, while the reverse is true for Davies-Bouldin index.

In Table 1 we can see the results of both tests on each of the clustering techniques, for LIME weights and SHAP values respectively. Both techniques assign a score to represent internal clusters cohesion and external distant from one another: the silhouette scores tells us the clusters are better defined as it advances in positive territory, whereas the Davies-Bouldin index dispersion is lower (and therefore clusters are better) the lower is the score.

**TABLE 1:** Clustering evaluation results.

Method	LIME	SHAP
K-means Silhouette	0.143	0.370
Spectral clustering Silhouette	0.141	0.370
K-means DBI	2.325	1.126
Spectral Clustering DBI	2.329	1.106

As it turns out, SHAP values seem to constitute an input space more suitable to be divided into clusters, with a clear advantage in discriminative power in this unsupervised setting. The measures we employed for this evaluation take into consideration the entire numerosity of dimensions, which in this case is 46 since we have one parameter for each of the original feature, whereas with a scatterplot we can only evaluate two dimensions at a time.

**FIGURE 2:**
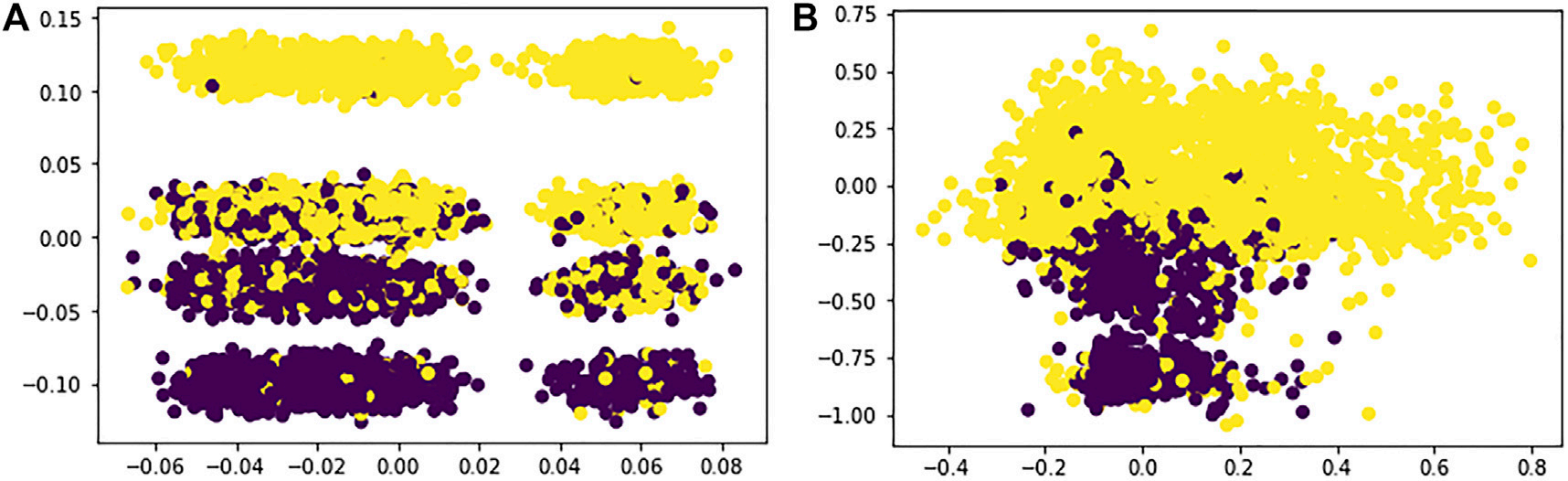
**(A)** Lime spectral clustering; **(B)** Shap spectral clustering.

For reference, in Figure 2 we report bidimensional plots for each case, where we can see how spectral clustering assigned each data point to the respective clusters by looking at the different colors; here, of course, we can only see this division across two dimensions, but we can already notice how SHAP value clustering seem to better divide the two clusters in space.

Having established the superiority of SHAP values in the unsupervised environment, we can now test the predictive power of both families of parameters. To this end, we run several Random Forest algorithms (Breiman, 2001) with optimized hyperparameters and compare the means of the Area under the Curve (AUC) (Bradley, 1997). We employ Random Forests to evaluate parameters’ preditive power because it has less hyperparameters to optimize, it better handles multicollinearity and it’s better parallelizable, thus allowing us to increase the number of runs significantly. Furthermore, we don’t need a specific supervised learning algorithm to evaluate this point, as long as we use the same for both sets of parameters.

**FIGURE 3:**
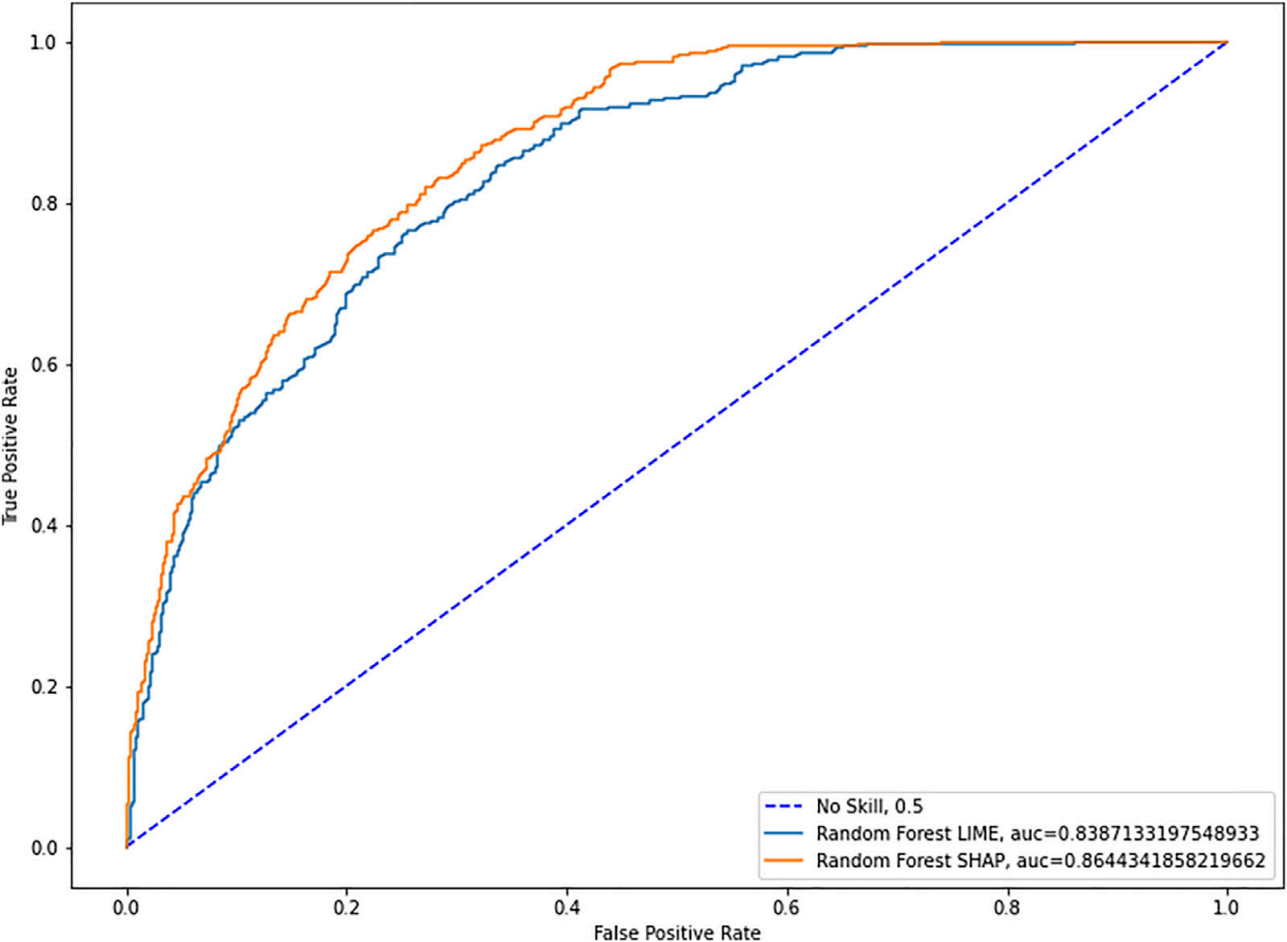
Lime and SHAP ROC curves.

As we can see in Figure 3, with a mean AUC of 0.864 for SHAP versus one of 0.839 for LIME and 50 repetitions, we find that the difference in means is statistically significant with a *p*-value of 0.0035.

Therefore, SHAP values appear to be better than Lime weights in assignign values to the dynamics of credit default as they are picked up by the XGBoost algorithm, dynamics upon which we looked for discriminative power, that is the objective of this paper.

## 4 Conclusion

The estimation of Probability of Default is a key element in the economic life of modern societies, and we now have the instruments and technologies to improve it significantly and lead away from the simplistic assumptions we used to follow in order to avoid undetected risks. This concretizes in an improve adherence to reality, were we have more dimensions available regarding the entity we want to evaluate and at the same time we are more capable and correct in such evaluation. We have already seen in the aforementioned works that the methodology based on a highly accurate predictive model combined with an interpretability tool allows us to reap the benefit of this improved precision without sacrificing explainability; our approach shows that some XAI models may be better than others and, furthermore, that elements coming from eXplainable AI models can be used to further improve methodologies and add value to data.

Some other works are already moving in this direction: see for instance (Bussmann, 2020; Bussman et al., 2021) or (Gramegna and Giudici, 2020) on the use of Shapley values to enrich the analysis and improve methods, but also (Giudici et al., 2020) and (Giudici and Raffinetti, 2020), with some innovative methodologies that combine well with XAI models.

Further research could find new ways to leverage the power of explanatory parameters and use them to deal with other issues concerning the Machine Learning pipeline, as well as extend the approach to other domains.

## Data Availability

The raw data supporting the conclusion of this article will be made available by the authors, without undue reservation.
